# Proteomics in IDH-mutated diffuse lower-grade glioma: a scoping review

**DOI:** 10.1093/noajnl/vdaf258

**Published:** 2025-12-13

**Authors:** Carl-Johan Kihlstedt, Anna Dénes, Alireza Mansouri, Nicholas Mikolajewicz, Thomas Skoglund, Linus Köster, Alba Corell, Helena Carén, Sandra Ferreyra Vega, Thomas Olsson Bontell, Annika Thorsell, Asgeir S Jakola

**Affiliations:** Department of Clinical Neuroscience, Institute of Neuroscience and Physiology at the Sahlgrenska Academy, University of Gothenburg, Gothenburg, Sweden; Department of Clinical Neuroscience, Institute of Neuroscience and Physiology at the Sahlgrenska Academy, University of Gothenburg, Gothenburg, Sweden; Penn State Cancer Institute, Penn State Health Milton S. Hershey Medical Center, Hershey, PA, USA; Department of Neurosurgery, University of Toronto, Toronto, Canada; Department of Clinical Neuroscience, Institute of Neuroscience and Physiology at the Sahlgrenska Academy, University of Gothenburg, Gothenburg, Sweden; Department of Neurosurgery, Region Västra Götaland, Sahlgrenska University Hospital, 41345 Gothenburg, Sweden; Department of Clinical Neuroscience, Institute of Neuroscience and Physiology at the Sahlgrenska Academy, University of Gothenburg, Gothenburg, Sweden; Department of Neurosurgery, Region Västra Götaland, Sahlgrenska University Hospital, 41345 Gothenburg, Sweden; Department of Clinical Neuroscience, Institute of Neuroscience and Physiology at the Sahlgrenska Academy, University of Gothenburg, Gothenburg, Sweden; Department of Neurosurgery, Region Västra Götaland, Sahlgrenska University Hospital, 41345 Gothenburg, Sweden; Sahlgrenska Center for Cancer Research, Department of Medical Biochemistry and Cell Biology, Institute of Biomedicine, Sahlgrenska Academy, University of Gothenburg, Gothenburg, Sweden; Sahlgrenska Center for Cancer Research, Department of Medical Biochemistry and Cell Biology, Institute of Biomedicine, Sahlgrenska Academy, University of Gothenburg, Gothenburg, Sweden; Department of Clinical Pathology, Sahlgrenska University Hospital, Gothenburg, Sweden; Department of Physiology, Institute of Neuroscience and Physiology, Sahlgrenska Academy, University of Gothenburg, Gothenburg, Sweden; Proteomics Core Facility, Sahlgrenska Academy, Gothenburg University, Sweden; Department of Clinical Neuroscience, Institute of Neuroscience and Physiology at the Sahlgrenska Academy, University of Gothenburg, Gothenburg, Sweden; Department of Neurosurgery, Region Västra Götaland, Sahlgrenska University Hospital, 41345 Gothenburg, Sweden

**Keywords:** astrocytoma, glioma, liquid chromatography-mass spectrometry, oligodendroglioma, proteomics

## Abstract

**Background:**

Therapeutic options and biomarkers for isocitrate dehydrogenase-mutated (IDHmut) diffuse lower-grade glioma (dLGG), WHO grade 2–3, are limited. Global quantitative proteomics has aided the discovery of novel markers and drug targets across various pathologies. This review aimed to summarize current proteomic findings in IDHmut dLGG.

**Methods:**

PubMed, Embase, and Scopus were searched following PRISMA-ScR guidelines. Studies examining quantitative proteomics in IDHmut dLGG with liquid chromatography–mass spectrometry in adult human samples were included. Studies with only high-grade gliomas, without IDHmut, using xenografts, or cell line samples, and reviews were excluded.

**Results:**

In total, 1,902 records were identified; 85 full-texts were retrieved, and 13 met the inclusion criteria. Twelve studies were cross-sectional and one longitudinal. Two studies used cerebrospinal fluid samples, while seven used fresh frozen and five formalin-fixed paraffin-embedded (FFPE) tissue samples. There was a large heterogeneity in aims, sample types, and analytical techniques. The most recurrent finding was altered energy metabolism, mostly related to the tricarboxylic acid cycle, compared to IDH-wildtype gliomas. IDHmut dLGG proteomic profile was distinct from other brain tumors, including IDH-wildtype glioblastoma, IDHmut grade 4 astrocytomas, and grade 1 gliomas or normal brain.

**Conclusions:**

IDHmut dLGG has a unique proteome that may be leveraged for biomarkers and therapeutic discovery. Proteomic findings indicate a particular dependency on glutamate metabolism to sustain the citric acid cycle and energy production. Although current proteomic knowledge is limited and fragmented, technological advancements present an opportunity for large-scale studies using FFPE samples, advancing proteomic knowledge and precision medicine in IDHmut dLGG.

Key Points

- IDH-mutated diffuse lower-grade gliomas have a distinct proteome that may be leveraged.
- Proteomic findings indicate a particular dependency on glutamate metabolism.
- Proteomic advances enable large-scale studies on well-annotated biobanked samples.

Importance of the Study

Quantitative global proteomics offers a powerful approach to aid in the discovery of new biomarkers and therapeutic targets. In IDH-mutated diffuse lower-grade glioma, available markers and therapeutic options remain limited, underscoring the importance of proteomic research. However, proteomic studies in IDH-mutated diffuse lower-grade glioma are scarce and fragmented. In this review, we synthesize current proteomic findings in IDH-mutated diffuse lower-grade glioma, highlighting key proteomic discrepancies compared to other brain tumors and normal brain tissue. Altogether, proteomic evidence indicates an altered energy metabolism and, more specifically, a particular dependency on glutamate. This observation is further supported by the overlap of differentially expressed proteins identified across several studies. Finally, we emphasize the potential for future large-scale studies leveraging archived formalin-fixed paraffin-embedded samples with detailed clinical data.

## Introduction

Molecular classification and introduction of targeted therapies are poised to significantly transform the management of diffuse lower-grade gliomas (dLGG). Isocitrate dehydrogenase mutations (IDHmut) are recognized as the hallmark of dLGG.^1^ These tumors are further classified into astrocytomas and oligodendrogliomas. Oligodendrogliomas, in addition to carrying an *IDH* mutation, also harbor chromosomal 1p/19q codeletions and are associated with a more favorable prognosis.^1^^,^^2^ The molecular aberration of IDHmut can be targeted, and treatment with *IDH* enzyme inhibitors is showing promising results in clinical trials.^3^ However, significant knowledge gaps and limited therapeutic options persist. Aside from the oncometabolite 2-hydroxyglutarate (2-HG), a tricarboxylic acid cycle (TCA) metabolite and promising but not yet validated biomarker of IDHmut gliomas,^4^ both subclass-specific prognostic proteomic markers and general predictive markers are lacking. Specific circulating plasma- or cerebrospinal fluid (CSF) biomarkers remain limited.

Compared to most other brain cancers, dLGG is associated with relatively longer survival, with a median survival of eight years in astrocytomas and 14 years in oligodendrogliomas.^5^ Following initial surgery, clinicians face the challenge of deciding when to initiate additional treatment, weighing potential survival benefits against the risk of long-term side effects. Reliable prognostic or treatment response prediction biomarkers would be invaluable in guiding these decisions. Identification of new therapeutic targets could also expand the treatment arsenal and be useful across different stages of the disease.

The proteome can be defined as a cell, tissue, or organism’s overall protein content at a specific time.^6^ Proteomics enhances our understanding of biological structure and function by reflecting the functional output of genes. However, since protein expression fluctuates over time and is influenced by environmental factors, the proteome is inherently more complex to study than the genome.^7^ Given that proteins frequently constitute the defining features of tumor phenotypes and the target of numerous drugs, proteomic research offers a promising avenue for discovering new biomarkers and therapeutic targets, thereby bridging existing gaps in disease understanding and management.^8^

Technological advancements in recent years, particularly in quantitative mass spectrometry (MS)-based approaches and the ability to analyze large-scale data, have accelerated proteomic research, resulting in the discovery of novel cancer biomarkers and drug targets.^9–11^ Nevertheless, proteomic discoveries in dLGG are limited and fractured. In this scoping review, we compile the current knowledge on IDHmut dLGG proteomics, highlight key findings, and propose future research directions.

## Methods

We conducted a scoping review to systematically map current research and identify existing knowledge gaps related to the proteomics of IDHmut dLGG. The following research question was formulated: What is known about the proteome from using quantitative proteomics in adult patients with IDHmut dLGG in the current literature?

### Literature search

A systematic literature review was conducted in accordance with PRISMA guidelines extensions for scoping reviews.^12^ PubMed MEDLINE (National Library of Medicine), Embase (Elsevier), and Scopus (Elsevier) databases were searched for studies conducting proteomics on IDHmut dLGG. No search restrictions were applied. Full search strings are available in Supplementary Material 1. After the screening process was completed, references and updated reviews were searched to identify any relevant articles that might have been missed in the initial search. No review protocol was registered.

### Screening

**Figure 1.**
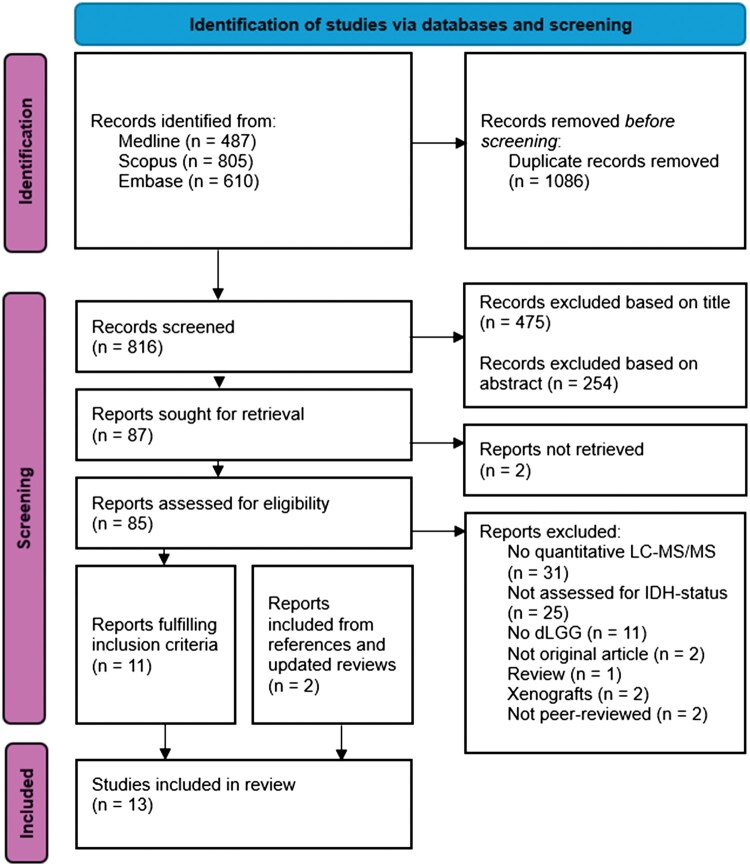
PRISMA flowchart detailing the identification and screening process. Flow diagram of the selection process. LC-MS/MS, liquid chromatography–tandem mass spectrometry; IDH, isocitrate dehydrogenase; dLGG, diffuse lower-grade gliomas.

Titles, then abstracts, then full texts were independently screened by two authors (CK and AD) to identify relevant studies (Figure 1). In case of disagreement between the screening authors, a third author (ASJ) served as an adjudicator. Inclusion criteria were as follows: peer-reviewed English-language articles with full-text availability, using global quantitative liquid chromatography–tandem mass spectrometry (LC-MS/MS) based methods on biological samples from adult (>18 years old) patients diagnosed with IDHmut dLGG (grade 2-3). Exclusion criteria included: studies analyzing only high-grade (grade 4) glioma samples, studies using only targeted LC-MS/MS, studies without IDHmut status determination, studies using xenografts or cell line samples, and review articles. No restrictions for year of publication or minimum sample size were applied.

### Data extraction

Data were extracted independently from the included articles using a standardized form (see Supplementary Table 1). Several authors evaluated the form before the data extraction process was initiated. Data charting was conducted independently. Variables extracted included: year of publication, study population, sample type and quantity, version of the WHO classification criteria for brain tumors used, proteomic assay and instrument, number of identified proteins, differentially expressed proteins (DEPs), differentiated protein profiles, enrichment analyses, and associated proteomic findings concerning IDHmut dLGG. No critical appraisal of the included studies’ quality was performed.

### Analysis of overlapping differentially expressed proteins

All collected lists of DEPs were converted to UniProt accession IDs. Lists of DEPs from similar comparisons were then screened for overlap using the web-based tool InteractiVenn.^13^ Clustering analysis was performed on overlapping DEPs with the Markov cluster algorithm using the STRING database web-based tool (version 12.0). Pathways associated with proteins in relevant clusters were extracted from Gene Ontology (GO), Kyoto Encyclopedia of Genes and Genomes (KEGG), Reactome, and WikiPathways databases within the same tool.

## Results

The selection process is outlined in Figure 1. The literature search was conducted on December 10th, 2024, with a supplementary search on June 16th, 2025. In total, 1,902 studies were identified. After removing duplicates and screening titles and abstracts for relevance, 85 unique studies were retrieved. Of these, 11 met al. inclusion criteria following full-text review. A search of references and updated reviews identified two additional studies that met the inclusion criteria, bringing the total number of studies included to 13.

### Characteristics of included studies

The characteristics of included studies are shown in Table 1, and a summary of all extracted data can be found in Supplementary Table 1. Most studies were recent; nine of the 13 were published after 2020, with the earliest in 2016. All but one study was cross-sectional;^14–25^ the remaining study was longitudinal, with samples collected at diagnosis and reoperation, separated by a median interval of 46 months.^26^ Two studies included only untreated, nonrecurrent samples.^16^^,^^25^ Four studies used a mix of treated/untreated and initial/recurrent samples.^14^^,^^18^^,^^19^^,^^21^ The remaining studies did not specify sample status. Five studies focused primarily on dLGG, while the rest focused on gliomas in general or glioblastoma (GBM), with dLGG as controls. The number of dLGG samples in each cohort ranged from 4 to 88. Five studies used the 2021 WHO classification, six used the 2016 version, and two used the 2007 criteria. Besides *IDH* mutational status, eight of the 13 studies also assessed 1p/19q co-deletion status. Nine studies made their MS data publicly available; links to directories are available in Supplementary Table 1.

**Table 1.** Characteristics of included studies

Study	Main research focus	Sample	Number of samples (n)	WHO classification	Mass spectrometry quantification method, instrument	Proteins identified
Salem et al., 2025	Identifying specific proteins and pathways for glioma subgroups using quantitative proteomics	FF tissue samples from diffuse gliomas	IDHmut dLGG (n = 34), IDHmut HGG (n = 12) GBM IDHwt (n = 36) (proneural GBM (n = 9), classical GBM (n = 14), mesenchymal GBM (n = 13))	2021	MS2 TMT, Q-Exactive HF Orbitrap	5,057
Zhang et al., 2024	Identifying proteomic classification for glioma and potential biomarkers	FFPE tissue samples from gliomas and the normal-appearing brain	grade 1 gliomas (n = 6) IDHmut AC (n = 51) (grade 2 (n = 27), grade 3 (n = 14), grade 4 (n = 9)) IDHmut ODG (n = 47) (grade 2 (n = 28), grade 3 (n = 19)) GBM (n = 82), gliomas not elsewhere classified (n = 3) normal-appearing brain (n = 32)	2021	LFQ 4D DIA, TimsTOFPro	8,561
Hudson et al., 2024	Identifying prognostic biomarkers associated with glioma progression	FF tissue samples from gliomas at initial diagnosis and later reoperation	IDHmut glioma (n = 6) at diagnosis: grade 2 (n = 4), grade 3 (n = 2) recurring as: grade 3 (n = 1), grade 4 (n = 5)	2021	DIA SWATH-MS, TripleToF 6600+	1,806
Yang et al., 2024	Distinguishing primary brain tumors and metastasis based on proteomics	FF tissue samples from gliomas and brain metastasis	gliomas (n = 14) (grade 2 IDHmut AC (n = 2), grade 3 IDHmut ODG (n = 1), grade 4 IDHmut AC (n = 4), grade 4 IDHwt (n = 7)) brain metastases (n = 15) (lung cancer (n = 10), breast cancer (n = 3), colorectal cancer (n = 1), ovarian cancer (n = 1))	2016	LFQ DDA, TimsTOF	8,165
Wang et al., 2023	Proteogenomic profiling of glioma tumors	FFPE tissue samples from gliomas and normal brain	grade 2 IDHmut ODG (n = 18), grade 2 IDHmut AC (n = 9), grade 3 IDHmut AC (n = 2), grade 4 IDHmut AC (n = 6) IDHwt GBM (n = 138), IDHmut GBM (n = 6), gliomas not elsewhere classified (n = 8), normal brain (n = 6)	2021	LFQ DDA, QExactive HFX or Orbitrap Exploris	16,675
Wong et al., 2022	Investigate transcriptomic and proteomic differences across LGG subtypes	FFPE tissue samples from dLGG and normal brain	IDHmut ODG (n = 21), IDHmut AC (n = 17), IDHwt (n = 10), normal brain (n = 6)	2021	MS3 TMT, Orbitrap Fusion Tribrid	5,894
Wang et al., 2020	Investigate differences in the proteome of GBM and LGG exosomes	Cells and exosomes isolated from CSF samples from patients with dLGG, GBM, and normal brain	grade 2/3 IDHmut AC (n = 4), grade 3 IDHmut ODG (n = 3) GBM IDHwt (n = 7) samples without CNS disease or tumor (n = 4)	2016	LFQ DDA, Q-Exactive hybrid quadrupole-orbitrap	2,958
Oh et al., 2020	Identifying biomarkers for GBM IDHwt based on phospho and global proteomics	FF tissue samples from GBM, dLGG, and normal brain	dLGG IDHwt (n = 5), dLGG IDHmut (n = 4) GBM IDHwt (n = 39), GBM IDHmut (n = 2) normal brains (n = 4)	2016	MS2 TMT, Q-Exactive	9,367
Dekker et al., 2020	Investigates changes in protein levels associated with *IDH* mutations in diffuse gliomas	FFPE tissue samples from diffuse gliomas	grade 2/3 IDHmut AC (n = 18), grade 2 IDHmut ODG (n = 11) GBM IDHwt (n = 19)	2016	LFQ DDA, and targeted PRM, Q Exactive HF	1,509
Djuric et al., 2019	Investigates proteomics of glioma subgroups	FF and macrodissected FFPE tissue samples from gliomas. FFPE tissue samples from meningiomas and medulloblastoma were used as controls.	FF samples (n = 15) (grade 1 (n = 3), grade 2/3 IDHmut ODG (n = 4), grade 2/3 IDHmut AC (n = 3), grade 4 IDHmut ODG (n = 2), grade 4 IDHmut AC (n = 1), grade 4 IDHwt (n = 2)) FFPE samples (n = 15) (grade 2/3 IDHmut ODG (n = 4), grade 2/3 IDHmut AC (n = 2), grade 4 IDHmut AC (n = 1), grade 4 IDHwt (n = 5), meningiomas (n = 2), medulloblastoma (n = 1))	2016	LFQ DDA, Q Exactive Plus	5,496
Dettling et al., 2018	Investigates potential T-cell antigens of IDHmut dLGG using proteomics	FF tissue from dLGG	IDHmut dLGG (n = 4)	2016	Identification only, LTQ Orbitrap XL	2,897
Gahoi et al., 2017	Investigates proteomic changes in CSF of different grades of gliomas	CSF samples from patients with gliomas and controls	Glioma (n = 17) (IDHmut grade 2 (n = 3), IDHmut grade 3 (n = 5) IDHwt grade 2 (n = 1), IDHwt grade 3 (n = 1), GBM (n = 7)) controls (n = 3)	2007	iTRAQ DDA, Q-TOF	∼170
Oktay et al., 2016	Investigates the genetic single-nucleotide polymorphism rs55705857’s role in glioma oncogenesis	FF tissue from grade 2 IDHmut gliomas	grade 2 IDHmut OG ATRXwt (n = 9) (3 A/G and 6 A/A) grade 2 IDHmut AC ATRXmut (n = 7) (3 A/G and 4 A/A)	2007	Not stated	1,561

Abbreviations: AC, astrocytoma; CSF, cerebrospinal fluid; CNS, central nervous system; DIA, data-independent acquisition; dLGG, diffuse lower-grade gliomas; FF, fresh frozen; FFPE, formalin-fixed paraffin-embedded; GBM, glioblastoma; HGG, high-grade gliomas; IDH, isocitrate dehydrogenase; IDHmut, IDH mutated; IDHwt, IDH wildtype; LFQ, label-free quantification; MS, mass spectrometry; ODG, oligodendroglioma; TMT, tandem mass tag.

### Proteomic Methodologies

Two studies used CSF samples,^18^^,^^24^ while the others analyzed tissue samples. Fresh frozen (FF) tissue was the most common sample type, used in seven studies,^19–23^^,^^26^ whereas five studies used formalin-fixed paraffin-embedded (FFPE) tissue samples.^15–17^^,^^25^ One study utilized both FF and FFPE samples (n = 15 each) from different patients and reported similar proteomic data between the two sample types.^14^ The number of detected proteins in the FFPE and FF sample groups was comparable (∼2,500 vs ∼2,900 proteins), with a significant overlap in detected proteins. However, the similar number of quantified proteins was unexpected given the known impact of fixation on protein recovery and yield. The most common quantification approach was label-free quantification using data-dependent acquisition (LFQ-DDA), applied in five studies, while tandem mass tag (TMT) chemical labeling was used in two. No clear pattern was observed in the choice of quantification method with respect to sample type.

The number of quantified proteins varied considerably across studies, ranging from approximately 170 to 2,958 in CSF samples and from 1,509 to 16,675 in tissue samples. Such variation is expected due to differences in sample material, methodology, instrumentation, and database search strategies. Notably, most tissue studies identified approximately 2,000–8,000 proteins, whereas Wang et al.^25^ reported over 16,000, nearly twice as many as the next highest study. The unusually high number likely reflects the use of a redundant database containing multiple entries for the same protein. This may not only have inflated the number of identifications but also expanded the search space, which increases the proportion of false positives. Eight studies reported DEPs (Supplementary Table 2) along with enriched pathways across various comparisons, including IDHmut dLGG and GBM/IDH-wildtype (IDHwt), high and lower-grade astrocytomas, recurrent IDHmut dLGG and oligodendrogliomas. These findings are summarized in Table 2. The most frequently used database for pathway analyses was GO, used in five studies, followed by KEGG, used in two studies.

**Table 2.** Studies with IDHmut gliomas that reported DEPs and enriched pathways

Study	Comparison (n)	No. DEPs	Cut-offs for DEPs	Pathway database	Enriched pathways in IDHmut dLGG	Validation
Salem et al., 2025	IDHmut dLGG (n = 34) vs Classic GBM (n = 14)	162	Used FC fitted with protein-wise linear models	GO and KEGG	Mitochondrial organization, lipid metabolism, protein metabolism	No validation in dLGG.
	IDHmut dLGG (n = 34) vs Proneural GBM (n = 9)	144			Mitochondrial organization, lipid metabolism, pentose phosphate pathway	
	IDHmut dLGG (n = 34) vs Mesenchymal GBM (n = 13)	28			Translation, lipid metabolism	
	IDHmut dLGG (n = 34) vs IDHmut HGG (n = 12)	1			Golgi organization, translation	
Zhang et al., 2024	IDHmut glioma (n = 87) vs IDHwt glioma (n = 81)	204	FC > 2, P < 0.01	IPA	Lipid metabolism, fatty acid transport, DNA biosynthetic process, IL-12 signaling pathway, and IL-6 signaling pathway	No validation in IDHmut glioma or LGA.
	IDHmut LGA (n = 42) vs grade 4 IDHmut AC (n = 9)	34	FC > 2, P < 0.05		The cell cycle control of chromosomal replication pathway	
Hudson et al., 2024	IDHmut dLGG (n = 6) vs matched recurrent higher-grade glioma (n = 6)	32	FC > 2, P < 0.05	Not stated	The TCA cycle and synaptic pathways	Based on a literature search, 5 DEPs were chosen and validated with IHC.
Yang et al., 2024	IDHmut dLGG (n = 3) vs grade 4 IDHmut AC (n = 4)	793*	FC > 2 or < 0.5	GO, Reactome, WikiPathways, and KEGG	Energy metabolism such as mitochondrion organization and microtubule-based transport	No validation.
Wang et al., 2023	IDHmut dLGG (n = 29) vs GBM (n = 144)	1,391^a^	FC > 2, P < 0.05	GO	Lipid oxidation, glutamine metabloic processes, glutamine transport, and GADPH metabolism	No validation in dLGG.
Wong et al., 2022	IDHmut LGA (n = 17) vs ODG (n = 21) or IDHwt dLGG (n = 10)	24 + 46	FC > 1.5, P < 0.05	GO	Chromatin remodeling	No validation.
	ODG (n = 21) vs IDHwt dLGG (n = 10) or IDHmut LGA (n = 17)	197 + 24			Cell-adhesion	
	IDHwt dLGG (n = 10) vs ODG (n = 21) or IDHmut LGA (n = 17)	46 + 197			Metabolic pathway	
	IDHmut LGA (n = 17) vs ODG (n = 21) and IDHwt dLGG (n = 10)	2			-	No validation.
	ODG (n = 21) vs IDHwt dLGG (n = 10) and IDHmut LGA (n = 17)	27			Brain development	
	IDHwt dLGG (n = 10) vs IDHmut LGA (n = 17) and ODG (n = 21)	25			Immune infiltration, inflammation, and wound healing^b^	
Dekker et al., 2020	IDHmut dLGG (n = 29) vs GBM (n = 19)	48^c^	FDR < 0.05	Not stated	Glycolysis, pentose phosphate pathway, TCA cycle, and glutaminolysis metabolic pathways. Largest differences in aerobic glycolysis and glutaminolysis	No validation.
Djuric et al., 2019	IDHmut (n = 6) vs GBM (n = 5)	287	FDR < 0.1	GO	Oxidative-induced senescence, noncoding RNA metabolism, class I HDACs, and cadherin signaling	No validation in IDHmut.
Gahoi et al., 2017^d^	IDHmut dLGG (n = 6) vs IDHwt (n = 9)	10	FC >1.2 or <−1.2, P < 0.1	Not stated	Oxidative stress and reactive oxygen species	No validation.

List of DEPs, where available, can be found in Supplementary Table 2. Abbreviations: AC, astrocytoma; DEPs, differentially expressed proteins; dLGG, diffuse lower-grade gliomas; FC, fold change; GBM, glioblastoma; GO, gene ontology; IDH, isocitrate dehydrogenase; IDHmut, IDH mutated; IDHwt, IDH wildtype; IPA, ingenuity pathway analysis; IHC, immunohistochemistry; KEGG, kyoto encyclopedia of genes and genomes; LGA, low-grade astrocytomas; ODG, oligodendroglioma; TCA, tricarboxylic acid cycle.

a List of DEPs not available.

b Pathway associated with IDHwt dLGG and not IDHmut dLGG.

c Protein data from targeted mass spectrometry.

d Study using CSF samples.

In the following sections, we describe proteomic differences observed in IDHmut dLGG. As critical appraisal was beyond the scope of this study and sample sizes varied considerably between studies, the sample sizes and tissue types are reported in parentheses alongside each reported finding. Only one study validated its IDHmut dLGG proteomic results using additional techniques.^26^ All studies employed LC–MS/MS, as this was an inclusion criterion; for specific quantification methods and instruments used, see Table 1.

### IDHmut versus glioblastoma

Protein profiles clustered differently between IDHmut gliomas (mostly dLGG) and GBM when compared in four studies (n = 15–180, FF and FFPE)^14^^,^^15^^,^^17^^,^^19^ on tissue samples and one study (n = 17) with CSF samples.^24^ Compared with GBM, IDHmut gliomas exhibited higher levels of proteins involved in oxidative stress, glutamate metabolism, mitochondrial organization, and lipid metabolism (Table 2).

**Figure 2.**
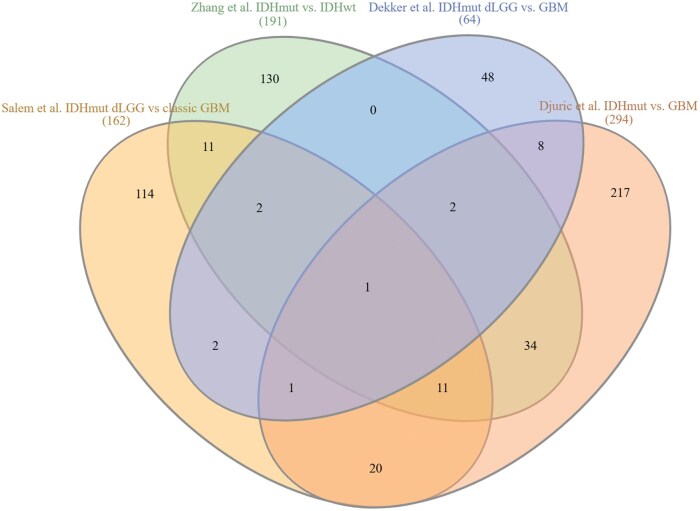
Overlapping DEPs in studies comparing IDHmut and GBM. Venn diagram of overlapping DEPs in studies comparing IDHmut and GBM. Lists of overlapping DEPs can be found in Supplementary Table 3. IDH, isocitrate dehydrogenase; IDHmut, IDH mutated; IDHwt, IDH wildtype; dLGG, diffuse lower-grade gliomas.

**Figure 3.**
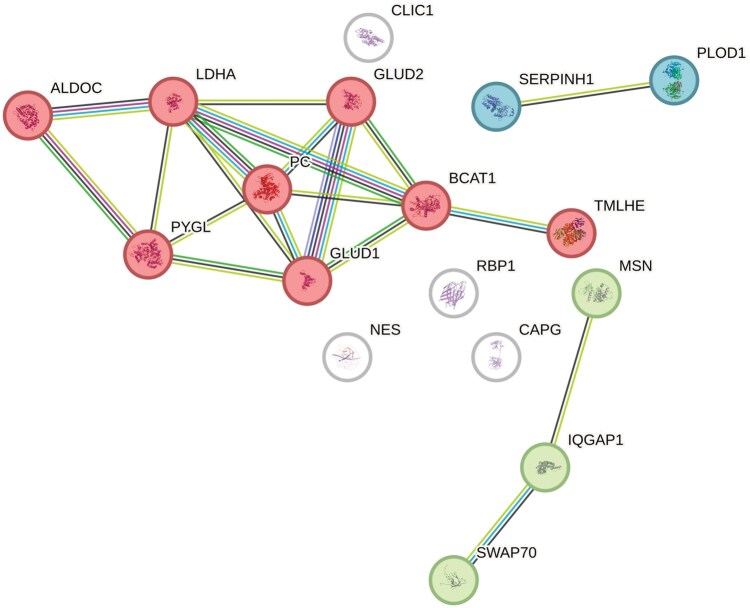
Cluster analysis of overlapping DEPs. STRING database web-based tool cluster analysis of 17 DEPs between IDHmut and IDHwt overlapping in three or four reviewed studies. Eight proteins (in red) form a cluster involved in the general metabolism of carbon and amino acids. GLUD1, GLUD2 and BCAT1 are specifically related to glutamate metabolism. Supporting data in Supplementary Table 4. IDH, isocitrate dehydrogenase; IDHmut, IDH mutated; IDHwt, IDH wildtype; DEPs, differentially expressed proteins.

**Figure 4.**
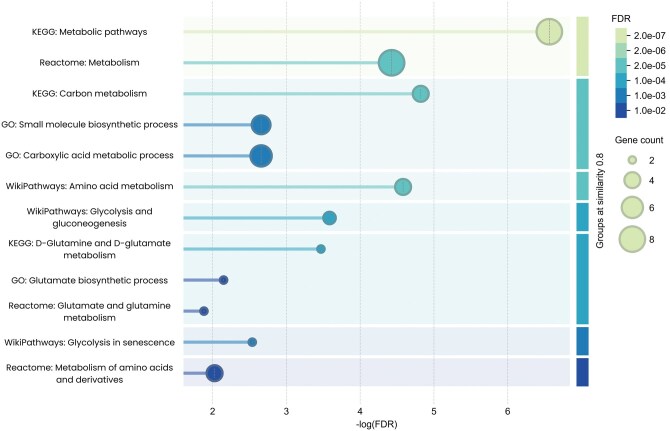
Extracted pathways associated with the identified metabolic cluster from overlapping DEPs, grouped after pathway similarity. STRING database web-based tool pathway analysis of the identified metabolic cluster of eight overlapping DEPs between IDHmut and IDHwt (Figure 3). Pathways are grouped after similarity. Showing the top three enriched pathways from gene ontology, kyoto encyclopedia of genes and genomes, Reactome, and WikiPathways databases. Supporting data in Supplementary Table 5. IDH, isocitrate dehydrogenase; IDHmut, IDH mutated; IDHwt, IDH wildtype; DEPs, differentially expressed proteins.

A list of DEPs was available from four studies. Screening for overlaps revealed 17 DEPs that were shared by at least three studies (Figure 2, Supplementary Table 3). Notably, one protein—ALDOC—was identified across all four studies. ALDOC is a glycolytic enzyme, and elevated expression has been associated with improved prognosis in GBM, while loss of ALDOC function has been suggested to promote tumor cell invasion and migration.^27^ Cluster analysis of the 17 DEPs identified a group of eight proteins related to metabolism (Figure 3, Supplementary Table 4). Extraction of associated pathways from databases revealed that the protein cluster was mainly involved in general carbon and amino acid metabolism, with three proteins—GLUD1, GLUD2, and BCAT1—specifically associated with glutamate metabolism (Figure 4, Supplementary Table 5). GLUD1 and GLUD2 (also known as GDH1 and GDH2) catalyze the conversion of glutamate to α-ketoglutarate, while BCAT1 initiates the catabolism of branched-chain amino acids by moving an amine group to α-ketoglutarate, producing glutamate. Upon closer inspection, four included studies (n = 15–180, FFPE) found that GLUD1 and GLUD2 were upregulated and BCAT1 downregulated in IDHmut gliomas compared to IDHwt gliomas.^14–17^

Related to this, in one included study (n = 48, FFPE), an exploratory MS analysis was followed by targeted MS with proteins associated with pathways identified in the initial screening. A total of 48 DEPs were identified between IDHmut dLGG and GBM, including GLUD1, GLUD2, and BCAT1. The largest expression differences were observed in enzymes and transporters involved in glutamate and lactate import and conversion into α-ketoglutarate.^15^ The authors concluded that IDHmut dLGG metabolism is rewired to increase glutamate and lactate import and their conversion into α-ketoglutarate, thereby decreasing metabolic stress induced by the mutation.

Interestingly, in one large study (n = 187, FFPE) with dLGG and GBM, gene analysis found *IDH1* and *EGFR* mutations (EGFRmut) to be mutually exclusive.^25^ In the same study, proteins involved in glutamate metabolism and the GABA receptor signaling pathway were upregulated in IDHmut and downregulated in EGFRmut compared to EGFR wildtype. Conversely, proteins associated with the PD-L1 signaling pathway and antigen processing and presentation were elevated in EGFRmut tumors and reduced in IDHmut tumors, relative to the respective wildtype.

### 1p/19q-codeletion

Three studies investigated the effects of 1p/19q co-deletion on the IDHmut dLGG proteome. In two of them (n = 29–38, FFPE), proteomic profiles of astrocytomas and oligodendrogliomas could not be reliably differentiated by 1p/19q status.^15^^,^^16^ Nevertheless, 24 DEPs were identified when comparing IDHmut astrocytomas with oligodendrogliomas in one study (n = 38, FFPE); pathway analysis revealed an enrichment of cell-adhesion related proteins in oligodendrogliomas, whereas chromatin remodeling pathways were enriched in IDHmut astrocytomas.^16^ Further, 54 subtype-specific proteins were identified across morphological lower-grade glioma subtypes (IDHwt, IDHmut astrocytoma/oligodendroglioma), such as Vimentin, Nestin, and BCAT1, which are recognized as potential glioma markers.^28–30^ BCAT1 is covered above as it was detected in our overlapping analysis. Nestin and Vimentin, both intermediate filament proteins, showed progressively increased expression from IDHmut oligodendrogliomas to astrocytomas to IDHwt gliomas. This pattern aligns with previous studies linking higher levels of these proteins to poorer patient survival.^29^^,^^30^ The third study analyzed both FFPE and FF samples (n = 7 vs 10); here, astrocytomas and oligodendrogliomas could be differentiated in FFPE samples but not in FF samples.^14^ No pathway analysis of DEPs related to 1p/19q-status was performed in this study.

### IDHmut dLGG versus IDHmut grade 4 astrocytomas

Yang et al. (n = 14, FF) found that IDHmut dLGG and IDHmut grade 4 astrocytomas have different proteomic signatures.^20^ DEPs identified in the comparison IDHmut dLGG and IDHmut grade 4 astrocytomas were linked to pathways, including lipid metabolism, cell cycle regulation, the TCA cycle, and mitochondrial organization (Table 2). Where available, DEPs were screened for overlap across studies; however, no overlapping DEPs were detected. A longitudinal study compared proteomics of six IDHmut dLGG at diagnosis and a later reoperation at which the disease stage had advanced (five grade 4 and one grade 3).^26^ Comparison of initial and recurrent tumor samples revealed 32 DEPs associated with the TCA cycle and synaptic pathways. Following a literature search to identify cancer-related associations, six proteins were selected (5 DEPs and one additional protein), and an immunohistochemistry analysis was performed. Three of the proteins–CA9, CYFIP2, and LGALS3BP–were associated with tumor progression. CA9 is a protein that is involved in pH regulation in hypoxic cancer cells; inhibition is known to increase the effectiveness of temozolomide in glioma cells.^31^ CYFIP2 is a pro-apoptotic protein associated with the actin skeleton, but how it relates to cancer is not well understood. Finally, LGALS3BP is a glycoprotein involved in both immune responses and various cancers.^32^ It is elevated in the serum of cancer patients and proposed as a biomarker for early detection in gliomas.^33^^,^^34^

### dLGG versus normal brain, grade 1 gliomas, and metastasis

Three studies (n = 54–139, FF and FFPE) identified different proteomic profiles between gliomas (in general) and either adjacent normal brain tissue or non-tumor brain controls, where gliomas had an abundance of proteins involved in cell proliferation and immune response.^16^^,^^17^^,^^21^ One of these studies (n = 54, FFPE) found that IDHmut dLGG in particular also had a distinct protein profile from normal brain.^16^ Specifically, IDHmut dLGG had elevated levels of IDH1 and EGFR (unspecified variant), but reduced levels of IDH2 compared to normal brain. The proteome of IDHmut gliomas also differed from that of grade 1 gliomas (n = 13, FF), which exhibited higher levels of proteins related to the extracellular matrix and reduced levels of proteins involved in glutamate and other neurotransmitter signaling compared to other gliomas.^14^ Finally, gliomas in general formed distinct clusters separate from brain metastases (n = 29, FF).^20^

## Discussion

This scoping review synthesizes current findings on global quantitative proteomics in IDHmut dLGG and explores their implications for future research and clinical translation. We identified 13 studies that applied quantitative LC-MS/MS proteomics to IDHmut dLGG, revealing substantial heterogeneity in research aims, sample populations, methodologies, instrumentation, and database search strategies. A relatively low number of overlapping findings was observed, which may be attributed to biological variability in the samples, differences in sample preparation (FF vs FFPE), and diverse quantification strategies employed. Conversely, the findings collectively support the existence of a distinct proteome in IDHmut dLGG, as the protein profiles were distinctive compared to normal brain, metastases, grade 1 gliomas, IDHmut grade 4 astrocytomas, and GBM.^14–16^^,^^20^^,^^21^^,^^24^ These findings support the hypothesis that the IDHmut dLGG proteome harbors unique features that could be leveraged to identify biomarkers or therapeutic targets. Two studies compared IDHmut dLGG with non-tumor brain tissues, but both were limited by small sample sizes and restricted analyses. Included studies instead mostly focused on comparisons with other tumors.

Most reviewed studies identified changes in energy metabolism, and three studies (n = 6–48, FF and FFPE) found alterations specifically in the TCA cycle in IDHmut dLGG.^15^^,^^20^^,^^26^ These findings were anticipated since the IDH enzyme catalyzes a key reaction in the TCA cycle, and align with previous findings.^35^ One study (n = 48, FFPE) found altered metabolic enzyme ratios, suggesting an increased uptake and conversion of lactate and glutamate into α-ketoglutarate, likely as a compensatory mechanism to maintain TCA cycle activity in IDHmut cells.^15^ Interestingly, our cluster analysis of overlapping DEPs between IDHmut and IDHwt identified three proteins associated with glutamate metabolism, GLUD1, GLUD2, and BCAT1. In four reviewed studies (n = 15–180, FFPE), GLUD1 and GLUD2 (also referred to as GDH1 and GDH2), which facilitate the conversion of glutamate to α-ketoglutarate, were consistently upregulated, whereas BCAT1, which catalyzes a reverse reaction from α-ketoglutarate to glutamate, was downregulated.^14–17^ These alterations further reinforce the hypothesis of glutamate metabolic rewiring in IDHmut gliomas. Altered metabolic patterns can also be observed in previous RNA expression data.^36^ If validated, this metabolic dependency could represent a novel therapeutic target for selectively impairing tumor metabolism in IDHmut dLGG. Supporting this concept, inhibition of glutaminase—an enzyme that converts glutamine to glutamate—has been shown to slow the growth of IDHmut gliomas and acute myeloid leukemia (AML).^37^ Furthermore, the oral glutaminase inhibitor CB-839 has demonstrated therapeutic efficacy in IDHmut AML, reducing 2-HG production and inducing differentiation.^38^ While similar results have not yet been reported in IDHmut gliomas, an ongoing phase I clinical trial is currently evaluating the combination of CB-839 with radiation and temozolomide in patients with IDHmut diffuse or anaplastic astrocytoma (NCT03528642).

The brain’s predominant excitatory neurotransmitter is glutamate, and elevated levels are associated with seizures in glioma patients.^39^ If intracellular glutamate levels rise, excessive glutamate is released via the cysteine/glutamate transporter Xc-.^40^ One included study (n = 187, FFPE) found that glutamate receptor signaling, neurotransmitter receptor transport, and regulation of GABA signaling were upregulated in IDHmut dLGG compared to GBM.^17^ Seizures are a common symptom in IDHmut dLGG and occur more frequently compared to GBM.^41^ If this is a result of the upregulated glutamate metabolism and receptors/transporters in IDHmut dLGG, future proteomic measurements of glutamate-associated protein biomarkers could potentially identify patients at elevated seizure risk.

Glutamate is also relevant to a phenomenon known as “synaptic hijacking”, where brain tumors, like glioma, form direct synaptic connections with healthy neurons and exploit the synaptic signals to facilitate tumor initiation, growth, and invasion.^42^ Such neurogliomal-synapses have been shown to increase glioma progression by stimulating growth and invasion.^43^ This process is mediated by the AMPA receptor, which makes AMPAR-inhibiting antiepileptic drugs an interesting choice for future clinical trials, building upon preliminary evidence from a small patient cohort and in xenografted mice.^44^^,^^45^

Apart from 2-HG, validated biomarkers in IDHmut dLGG remain limited. All studies identified DEPs to some extent, though these varied depending on the comparison groups used and thresholds applied. Several studies performed enrichment analysis to identify pathways and biological processes of interest, but did not further investigate the individual proteins driving these enrichments or their specific roles within the pathways. However, Hudson et al., the only longitudinal study (n = 6, FF) reviewed, reported that the abundance of CA9, CYFIP2, and LGALS3BP increased with tumor grade, suggesting their potential involvement in glioma progression.^26^ Most notable is CA9, an enzyme that helps the cell maintain normal pH in acidic environments, a key adaptation to hypoxia.^46^ It is regulated by pathways related to treatment resistance (HIF-1, MAPK, PI3K),^26^ and inhibition of the protein is shown to enhance temozolomide efficacy in GBM-bearing mice.^47^ Further, CA9 has previously been linked to poor prognosis in other cancers, as well as gliomas.^48–52^ Although none of the three proteins reached statistical significance as standalone biomarkers, a panel including all three was able to distinguish between long and short survival (area under the curve = 0.75). Before clinical integration, further validation and pathological analysis of these promising results are needed.

Biomarker panels are generally preferred over single-marker assays, as they offer greater robustness and clinical relevance. Once validated, such panels can be implemented using immunohistochemistry, making them accessible to most clinical laboratories. Such a panel could potentially be developed for IDHmut dLGG management. For instance, combining two of the previously mentioned proteins, CA9 (associated with hypoxia adaptation) and LGALS3BP (involved in immune modulation), along with the above-mentioned metabolic enzymes (e.g., TCA- or glutamate-associated enzymes), could potentially yield a robust signature for risk stratification in IDHmut dLGG subtypes. Moreover, protein-based biomarker panels may outperform genomic profiling due to their lower cost and broader applicability, or they could be combined with genomic data to improve the identification of patients most likely to benefit from IDH inhibitors. While these are promising prospects, extensive functional analyses and rigorous validation are required before clinical implementation.

Apart from the work of Hudson et al.^26^ outlined above, we found no other longitudinal proteomic studies and thus no further findings related to malignancy progression. As IDHmut dLGG is a disease with prolonged survival, often exceeding 10 years after diagnosis,^5^ there is a pressing need for insights into the mechanisms of tumor resistance and progression to help develop better monitoring techniques. This need will likely be exacerbated by the introduction of IDHmut inhibitors. Tumor tissue samples offer the highest specificity and molecular resolution, as they directly contain tumor cells and their microenvironment. However, tissue collection is invasive, limited to surgical or biopsy procedures, and may yield small or nonrepresentative samples, especially in heterogeneous tumors. Preferably, monitoring techniques should be of limited invasiveness. CSF provides such an alternative, as it represents a snapshot of all intracranial tissue, including the entire tumor. Mutations or other discrepancies that are absent in a spatial tissue sample may sometimes still be detectable in CSF.^53^ Nevertheless, tumor-derived molecules in CSF occur at low concentrations, and their detectability depends on tumor size and proximity to CSF spaces. Despite these limitations, CSF should be considered a valuable complement to tissue biopsies and a potential monitoring tool in neuro-oncology proteomics.

Only two of the included studies used CSF samples for their proteomic analysis; one (n = 18) did not disclose any IDHmut dLGG-specific findings,^18^ while the other (n = 20) found the IDHmut CSF proteome to be distinctive from IDHwt.^24^ In addition to this, earlier studies using other techniques outside the focus of this review (eg, western blot, immunohistochemistry, ELISA) have found several proteins with altered levels in IDHmut dLGG using CSF.^53–55^ Altogether, these findings suggest that the CSF proteome of IDHmut dLGG patients has measurable differences that could be monitored using liquid biopsy-based CSF proteomics, highlighting the potential for longitudinal proteomic studies using CSF samples. The results could shift proteomics from descriptive to predictive and complement existing tools like MRI for personalized wait-and-scan strategies.

Wong et al. (n = 48, FFPE) compared dLGG astrocytomas, oligodendrogliomas, and IDHwt samples and found 54 subtype-specific DEPs.^16^ In the dLGG astrocytoma group, only two subtype-specific DEPs were found, compared to 27 in oligodendroglioma and 25 in the dLGG IDHwt groups. This suggests greater heterogeneity within the astrocytoma group, which was further supported by unsupervised clustering, where astrocytomas were distributed among oligodendroglioma and IDHwt clusters instead of forming their own distinct cluster. These observations align with previous findings that astrocytomas are typically associated with a higher risk of malignant transformation and show greater morphological similarity than oligodendrogliomas to GBM.^1^^,^^56^ Notably, astrocytomas and oligodendrogliomas could not be segregated based on the proteome in any of the three studies (n = 6–21) using FF samples.^14–16^ However, one study (n = 4) achieved this separation when FFPE samples were used.^14^ This does not appear to be a consistent pattern, as another proteomic study (n = 11) on grade 3/4 gliomas using FFPE samples was also unable to differentiate 1p/19q status.^57^ Despite the limited sample sizes of the available studies, the collective findings suggest that 1p/19q status exerts a smaller influence on the dLGG proteome compared to the *IDH* mutation.

Most included studies used bulk proteomics, which presents a challenge of biomarker source validation, as cancer tissue harbors extensive cellular heterogeneity.^58^ This problem further extends to ill-defined “normal brain tissue” or other controls used, creating uncertainty at both ends of the comparison. Methods are needed to discern whether a marker represents dLGG, neurons, immune cells, blood vessels, etc. Spatial proteomics can help address this challenge by preserving the spatial context of protein expression within tissue sections, thereby enhancing biological interpretation and diagnostic accuracy. One method for achieving this is laser capture microdissection (LCM) to isolate specific cells or regions from a tissue sample, followed by LC-MS/MS.^59^ Another emerging method is to combine MS with imaging mass cytometry to reveal associations from bulk proteomic data with specific cancer or immune cells.^60^

Technological advancements in proteomics have opened up completely new opportunities in clinical research.^9^^,^^61^ Modern mass spectrometers using data-independent acquisition (DIA-MS) enable sensitive, high-throughput quantification of 10,000s of proteins. Quantifying large numbers of proteins is crucial for identifying biological mechanisms and for revealing disease-related pathways. Consequently, large-scale proteomic and multiomics initiatives, such as the *Clinical Proteomic Tumor Analysis Consortium* (CPTAC) and integrative data consortia like the *Molecular Tumor Board*,^62^ have been established to integrate and facilitate the clinical use of proteomic data.

Although FF tissue remains the gold standard due to its high proteomic depth, FFPE samples are far more accessible and often linked to rich clinical data, making them a valuable resource.^63^ Although proteomic studies on FFPE samples have historically faced challenges,^64–66^ recent advances in proteomic sample preparation and MS instrumentation have significantly improved protein recovery and quantification. The development of automated high-throughput platforms in clinical proteomics has the potential to enable large-scale, consistent protein analysis; accelerate biomarker discovery, and support precision medicine with more accurate diagnostic and personalized treatment strategies. For instance, recently introduced IDH inhibitors have been shown to prolong progression-free survival in patients with IDHmut dLGG.^67^ As novel therapeutic options continue to emerge, the need for reliable biomarkers becomes increasingly important, not only for disease monitoring but also for distinguishing responders from non-responders. In this context, proteomics holds significant promise as a powerful tool for biomarker discovery.

### Strengths and limitations

The main limitation of this review is that it covers an array of studies conducted across different years, using varying grade definitions, methods, and research questions, which limited direct comparisons and synthesis. On the other hand, the broad search criteria gave us the opportunity to evaluate the current state of proteomic knowledge in IDHmut dLGG, which represents both the study’s primary objective and its greatest strength. Such variability was anticipated to some extent, as this is an emerging field and ideal for a scoping review, which is why the format was chosen over a meta-analysis or systematic review. Finally, no critical appraisal or bias assessment was performed.

## Conclusion

The current state of proteomic research in IDHmut dLGG remains limited and fragmented. Although some cornerstones have been laid with discoveries of distinct protein profiles and metabolic associations, including a possible reliance on glutamate metabolism, a comprehensive mapping of the proteomic landscape and its divergence from other brain tumors and normal brain tissue is still lacking. Technological and analytical innovations offer significant potential to advance proteomic knowledge in IDHmut dLGG in the coming years. Multiomics approaches that integrate genomics, transcriptomics, metabolomics, and proteomics are increasingly used to identify novel biomarker panels and develop more sensitive and accurate diagnostic tools. When combined with clinical data, these multiomics datasets can enable data-driven decision-making and support precision medicine. To facilitate clinical translation, larger datasets derived from well-annotated, multi-center cohorts will be essential.

## Supplementary Material

vdaf258_Supplementary_Data

## Data Availability

All data used in this study are available in the supplementary materials.
