# Supplementary material for: Proteomics in IDH-mutated diffuse lower-grade glioma: a scoping review
Source: Neurooncol Adv. 2025 Dec 13;8(1):vdaf258. doi: 10.1093/noajnl/vdaf258 (PMC12932947; doi:10.1093/noajnl/vdaf258)
Supplement: vdaf258_Supplementary_Data [file vdaf258_supplementary_data.zip › Supplementary material 1.docx]

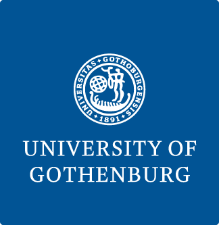

Biomedical Library, Gothenburg University Library

**Documentation of search strategies**

Date: 2024-12-10

Topic/research question: *protemik hos diffuse low grade gliomas (DLGG)*

Name of researchers: Carl Johan Kihlstedt

Librarians: Eva Hessman, Helen Sjöblom

Databases: Embase, Medline, Scopus

Total number of hits:

Before deduplication: 1678
After deduplication: 765

Updated search:

Date: 2025-06-18
Additional number of hits: 224 (Scopus n=85, Medline n=61, Embase n=78)

Scopus

| Interface: Elsevier  Date of Search: 2024-12-10  Number of hits: 720  Comment: | Field labels   - TITLE = title - ABS = abstract - KEY = keywords - W/x = within x words, regardless of order - * = truncation of word for alternate endings |
| --- | --- |
| \| **#** \| **Searches** \| **Results** \| \| --- \| --- \| --- \| \| 12 \| Limit 11 to english language \| 720 \| \| 11 \| 9 AND 10 \| 748 \| \| 10 \| TITLE-ABS-KEY(proteom* OR phosphoproteom* OR proteogenomic* OR “protein profiling” OR “protein mapping” OR “protein analysis” OR “protein quanti*” OR “relative quanti*” OR “absolute quanti*” OR “tandem mass tags” OR TMT OR “label free quanti*” OR “mass spectr*” OR MS OR LFQ OR “label-free LCMS” OR “label-free LC-MS” OR “data-independent acquisition” OR DIA OR “liquid chromatography-mass spectrometry” OR LC-MS OR LCMS OR “different* expressed protein*” OR DEPs OR DEP OR “selected reaction monitoring” OR SRM OR “multiple reaction monitoring” OR MRM OR “parallel reaction monitoring” OR PRM OR “stable isotope labeling by amino acids in cell culture” OR SILAC OR (“isobaric tags for relative” W/1 “absolute quantification”) OR iTRAQ) \| 1956375 \| \| 9 \| 5 OR 8 \| 19111 \| \| 8 \| 6 OR 7 \| 8128 \| \| 7 \| TITLE-ABS-KEY (IDHmut* OR DLGG OR LGG) \| 5003 \| \| 6 \| TITLE-ABS-KEY ( IDH W/3 muta* ) \| 3473 \| \| 5 \| 3 AND 4 \| 14061 \| \| 4 \| TITLE-ABS-KEY ( glioma* OR astrocytoma* OR oligodendr* ) \| 177006 \| \| 3 \| 1 OR 2 \| 169216 \| \| 2 \| TITLE-ABS-KEY ( "grade 2" OR "grade two" ) \| 39094 \| \| 1 \| TITLE-ABS-KEY ( ( low OR lower ) W/3 grade* ) \| 132699 \| | |

Medline
Ovid MEDLINE(R) and In-Process, In-Data-Review & Other Non-Indexed Citations

| Interface: Ovid  Date of Search: 2024-12-10  Number of hits: 426  Comment: In Ovid, two or more words are automatically searched as phrases; i.e. no quotation marks are needed | Field labels   - exp/ = exploded MeSH term - / = non exploded MeSH term - .ti,ab,kf. = title, abstract and author keywords - adjx = within x words, regardless of order - * = truncation of word for alternate endings - /FREQ=n  occurrence of a term, records only retrieved if  term/s occur at least n number of times |
| --- | --- |
| \| **#** \| **Searches** \| **Results** \| \| --- \| --- \| --- \| \| 13 \| limit 15 to english language \| 426 \| \| 15 \| 11 and 14 \| 434 \| \| 14 \| 12 or 13 \| 875785 \| \| 13 \| (proteom* or phosphoproteom* OR proteogenomic* OR protein profiling OR protein mapping or protein analysis or protein quanti* or relative quanti* or absolute quanti* or tandem mass tags or TMT or label free quanti* or mass spectr* or MS or LFQ or label-free LCMS or label-free LC-MS or data-independent acquisition or DIA or liquid chromatography-mass spectrometry or LC-MS MS or LC-MS or LCMS or different* expressed protein* or DEPs OR DEP or selected reaction monitoring  or SRM or multiple reaction monitoring or MRM or parallel reaction monitoring or PRM or stable isotope labeling by amino acids in cell culture or SILAC or (isobaric tags for relative adj1 absolute quantification) or iTRAQ).ti,ab,kf \| 809139 \| \| 12 \| Proteome/ or exp Proteomics/ or exp Mass Spectrometry/ \| 364503 \| \| 11 \| 7 or 10 \| 16027 \| \| 10 \| 8 or 9 \| 6532 \| \| 9 \| (IDHmut* OR DLGG OR LGG).ti,ab,kf \| 3723 \| \| 8 \| (IDH adj3 muta*).ti,ab,kf \| 3127 \| \| 7 \| 3 and 6 \| 12247 \| \| 6 \| 4 or 5 \| 160058 \| \| 5 \| exp Glioma/ \| 104594 \| \| 4 \| (glioma* OR astrocytoma* OR oligodendr*).ti,ab,kf \| 117102 \| \| 3 \| 1 or 2 \| 111997 \| \| 2 \| (grade 2 or grade two).ti,ab,kf \| 32291 \| \| 1 \| ((low or lower) adj3 grade*).ti,ab,kf \| 81574 \| | |

Embase

| Interface: Elsevier  Date of Search: 2024-12-10  Number of hits: 532  Comment: use ' for phrase searching | Field labels   - /exp = exploded Emtree term - /de = non exploded Emtree term - :ti,ab,kw. = title, abstract and author keywords - NEAR/n = within x words, regardless of order - * = truncation of word for alternate endings - * = variable wildcard, i.e one or more letters - ' = phrase |
| --- | --- |
| \| **#** \| **Searches** \| **Results** \| \| --- \| --- \| --- \| \| 14 \| #16 AND ('article'/it OR 'article in press'/it OR 'erratum'/it OR 'preprint'/it OR 'review'/it) \| 532 \| \| 16 \| limit 15 to english language \| 991 \| \| 15 \| 11 AND 14 \| 1011 \| \| 14 \| 12 OR 13 \| 1275377 \| \| 13 \| 'proteome'/exp OR 'proteomics'/exp OR 'mass spectrometry'/exp \| 787658 \| \| 12 \| (proteom* OR phosphoproteom* OR proteogenomic* OR 'protein profiling’ OR ‘protein mapping’ OR ‘protein analysis’ OR ‘protein quanti*' OR ‘relative quanti*’ OR ‘absolute quanti*’ OR ‘tandem mass tags’ OR TMT OR ‘label free quanti*’ OR ‘mass spectr*’ OR MS OR LFQ OR ‘label-free LCMS’ OR ‘label-free LC-MS’ OR ‘data-independent acquisition’ OR DIA OR ‘liquid chromatography-mass spectrometry’ OR LC-MS OR LCMS OR ‘different* expressed protein*’ OR DEPs OR DEP OR ‘selected reaction monitoring’ OR SRM OR ‘multiple reaction monitoring’ OR MRM OR ‘parallel reaction monitoring’ OR PRM OR ‘stable isotope labeling by amino acids in cell culture’ OR SILAC OR (‘isobaric tags for relative’ NEAR/1 ‘absolute quantification’) OR iTRAQ):ti,ab,kw \| 1069824 \| \| 11 \| 7 OR 10 \| 26596 \| \| 10 \| 8 OR 9 \| 11423 \| \| 9 \| (IDHmut* OR DLGG OR LGG):ti,ab,kw \| 7940 \| \| 8 \| (IDH NEAR/3 muta*):ti,ab,kw \| 6012 \| \| 7 \| 3 AND 6 \| 19858 \| \| 6 \| 4 OR 5 \| 255905 \| \| 5 \| 'glioma'/exp \| 195025 \| \| 4 \| (glioma* OR astrocytoma* OR oligodendr*):ti,ab,kw \| 165875 \| \| 3 \| 1 or 2 \| 190740 \| \| 2 \| (‘grade 2’ OR ‘grade two’):ti,ab,kw \| 67840 \| \| 1 \| ((low or lower) NEAR/3 grade*):ti,ab,kw \| 126764 \| | |
